# Dynamic Autophosphorylation of Mps1 Kinase Is Required for Faithful Mitotic Progression

**DOI:** 10.1371/journal.pone.0104723

**Published:** 2014-09-29

**Authors:** Xinghui Wang, Huijuan Yu, Leilei Xu, Tongge Zhu, Fan Zheng, Chuanhai Fu, Zhiyong Wang, Zhen Dou

**Affiliations:** 1 Hefei National Laboratory of Physical Sciences at the Microscale, University of Science and Technology of China, Hefei, China; 2 Anhui key Laboratory of Cellular Dynamics and Chemical Biology, University of Science and Technology of China, Hefei, China; 3 Department of Biochemistry, The University of Hong Kong, Hong Kong, China; 4 HKU-Shenzhen Institute of Research and Innovation, The University of Hong Kong, Shenzhen, China; 5 School of Chemistry and Materials Science, University of Science and Technology of China, Hefei, China; Huazhong University of Science and Technology, China

## Abstract

The spindle assembly checkpoint (SAC) is a surveillance mechanism monitoring cell cycle progression, thus ensuring accurate chromosome segregation. The conserved mitotic kinase Mps1 is a key component of the SAC. The human Mps1 exhibits comprehensive phosphorylation during mitosis. However, the related biological relevance is largely unknown. Here, we demonstrate that 8 autophosphorylation sites within the N-terminus of Mps1, outside of the catalytic domain, are involved in regulating Mps1 kinetochore localization. The phospho-mimicking mutant of the 8 autophosphorylation sites impairs Mps1 localization to kinetochore and also affects the kinetochore recruitment of BubR1 and Mad2, two key SAC effectors, subsequently leading to chromosome segregation errors. Interestingly, the non-phosphorylatable mutant of the 8 autophosphorylation sites enhances Mps1 kinetochore localization and delays anaphase onset. We further show that the Mps1 phospho-mimicking and non-phosphorylatable mutants do not affect metaphase chromosome congression. Thus, our results highlight the importance of dynamic autophosphorylation of Mps1 in regulating accurate chromosome segregation and ensuring proper mitotic progression.

## Introduction

The faithful distribution of duplicated genome into two daughter cells is governed by the spindle assembly checkpoint (SAC), requiring multiple mitotic kinases including CDK1, PLK1, Aurora A/B, Mps1, Bub1 and BubR1 [1]–[3]. Among these kinases, Aurora B, Mps1, Bub1 and BubR1 are involved in SAC signaling to halt mitosis at metaphase until all chromosomes are properly bi-orientated [4]–[5]. Until the SAC is satisfied, the anaphase promoting complex/cyclosome (APC/C) activator Cdc20 forms mitotic checkpoint complex (MCC) with other core SAC proteins Mad2, Mad3/BubR1 and Bub3 to inhibit the E3 ligase activity of APC/C, thus enabling to temporally halt mitosis before anaphase [6]–[7].

*Mps1* (*monopolar spindle 1*) was originally identified in budding yeast as a gene required for spindle pole body (SPB) duplication [8]. Subsequently, Mps1 orthologues were found in different species from fungi to mammals. Strikingly, the role of Mps1 in the SAC is well conserved through evolution. The human Mps1 protein (also known as TTK) displays maximum expression and kinase activity in mitosis and exhibits dynamic subcellular localization throughout mitosis [9]–[10]. In the absence of Mps1, the SAC is compromised [9], [11]. It is likely that Mps1 executes its function by recruiting Mad1 and Mad2 to unattached kinetochores. In addition to the SAC function, Mps1 was shown to contribute to the correction of improper kinetochore-microtubule attachments in different species [12]–[17].

Recently, the employment of small molecular inhibitors of Mps1 allows four groups to dissect the function of the human Mps1 independently [12]–[13], [16], [18]. Their chemical biology studies confirmed an indispensable role of Mps1 kinase activity in the SAC. In addition, these studies show that the kinase activity of Mps1 is required for the proper chromosome congression and accurate chromosome segregation. However, how Mps1 does so remains unclear. One model that Mps1 enhances Aurora B activity to promote proper chromosome congression and segregation has gained much attention, but still controversial [15], [19]. In contrast, the mechanistic detail as to how Mps1 is involved in the SAC has become much clearer recently. Several studies show that Mps1 phosphorylates its newly identified substrate KNL1 to promote the recruitment of Bub1 and Bub3 to kinetochore, thus maintaining robust SAC signaling [20]–[22].

The localization of Mps1 to kinetochore is important for its SAC function. Previous studies have mapped the kinetochore targeting domain of Mps1 to its N-terminal region (1–303 amino acids) [11], [23]. Interestingly, structural studies show that this N-terminal region contains a TPR domain (55–210 amino acids), highly similar to the TPR domains of Bub1 and BubR1 [24]–[25]. The kinetochore localization of Mps1 requires Aurora B kinase activity [16], [26]. Further, we demonstrated that Aurora B phosphorylates Hec1 to promote Mps1 localization to kinetochore [27]. As inhibition of Mps1 kinase activity significantly enhances the kinetochore localization of Mps1 [12], [16], [28], the Aurora B kinase activity may not be the sole contributor for the kinetochore localization of Mps1. It is likely that Mps1 itself is another contributing factor.

Mps1 undergoes autophosphorylation during mitosis [26], [29]–[31]. For example, autophosphorylation on Thr676 within the activation loop of Mps1 occurs in mitosis and this phosphorylation is required for its kinase activity *in vitro* and for the SAC *in vivo* [32]–[33]. Inhibition of the phosphorylation on Thr676 residue weakens the SAC and results in chromosomal instability without affecting cell viability [30]. Despite this progress, it is believed that more residues in Mps1 are autophosphorylated.

In this study, we first demonstrate that the previously identified autophosphorylation sites Thr12 and Ser15 are dispensable for Mps1 kinetochore localization. Further, we show that 8 other autophosphorylation sites are involved in regulating the kinetochore localization of Mps1. Our findings suggest that dynamic phosphorylation of Mps1 is essential for faithful chromosome segregation during mitosis.

## Materials and Methods

### Cell culture and drug treatments

HeLa cells were routinely maintained in DMEM (Invitrogen) supplemented with 10% FBS and penicillin-streptomycin (100 IU/ml and 100 mg/ml, respectively, GIBCO). BAC TransgeneOmics LAP-Mps1 stable cell line was kindly provided by Dr. Hyman and was maintained in the DMEM medium plus 0.5 µg/µl G418 [34]. Thymidine was used at 2 mM, Nocodazole at 100 ng/ml, Eg5 inhibitor STLC at 10 µM, Mps1 inhibitor Reversine at 0.5 µM, and MG132 at 20 µM.

### Plasmids and transfection

Wild type and kinase dead LAP-Mps1 and Mps1 shRNA constructs were described previously [15]. GFP-tagged Mps1 truncations Mps1^1–303^ and Mps1^1–524^ was generated by inserting the corresponding PCR-amplified fragments into pEGFP-C1 vector at BglII and SalI sites. Mutagenesis was performed using QuickChange site-directed mutagenesis kit (Stratagene) according to the manufacturer's instructions. All constructs were verified by sequencing. All the plasmids and siRNAs were transfected into cells using Lipofectamine 2000 (Invitrogen). To enrich mitotic cells, 12 hours after transfection, cells were treated with Thymidine for 14–16 hours, followed by release into normal DMEM medium. At 8 hours after release, cells were either treated with Eg5 motor inhibitor STLC or Monastrol for 2 hours and then were fixed for immunofluorescence staining. For rescue experiments, Mps1 shRNA was co-transfected with different rescue plasmids (or empty vector) at a 3∶1 ratio.

### Antibodies

Monoclonal anti-hMps1-N1 [9], anti-BubR1 [35], anti-Mad1 and anti-Mad2 [36] antibodies were used as previously described. Anti-α-tubulin (DM1A, Sigma) and ACA (Immunovision, Springdale, AR) were obtained commercially. For western blotting, HRP-conjugated anti-mouse or anti-rabbit antibodies (Pierce) were used.

### Kinase assays

Mps1 kinases were immunoprecipitated from 293T cell expressing different LAP-tagged Mps1 constructs. Recombinant maltose-binding protein (MBP) tagged Borealin and recombinant GST-Mps1were purified as previously described [26], [37]–[38]. *In vitro* phosphorylation assays were carried out at 30°C using immunopurified Mps1 kinase in 40 µl of kinase reaction buffer (50 mM Tris-HCl pH 7.5, 10 mM MgCl_2_, 0.5 mM DTT, 10 µM ATP, 5 µCi γ-^32^P-ATP). Reactions were stopped after 30 minutes by addition of SDS sample buffer. Samples were then resolved by SDS-PAGE and visualized by autoradiography.

### Immunofluorescence microscopy, image processing and quantification

HeLa cells grown on coverslips were fixed and permeabilized simultaneously with PTEMF buffer (50 mM PIPES, pH 6.8, 0.2% Trition X-100, 10 mM EGTA, 1 mM MgCl_2_, 4% Formaldehyde) at room temperature and were processed for indirect immunofluorescence microscopy. Samples were examined on a Deltavision microscope (Applied Precision), with optical sections acquired 0.2 µm apart in the Z-axis. Deconvolved images from each focal plane were projected into a single picture using Softworx (Applied Precision). In some case, images were collected using an Axioskop-2 with a 63× Plan Apochromat oil immersion objective of NA 1.4 (Zeiss). Images were taken at identical exposure times within each experiment, acquired as 24-bit RGB images, and processed in Adobe Photoshop. Images shown in the same panel have been identically scaled. Measurement of kinetochore intensities was performed in ImageJ (http://rsb.info.nih.gov/ij/) on non-deconvolved images. Quantification of kinetochore intensities was performed as previously described [39]. In brief, a circular region with fixed diameter was centered on each kinetochore, and unless indicated otherwise, anti-centromere antibody (ACA) intensity was measured in the same region and used for normalization (after subtraction of background intensity). The average pixel intensities from at least 50 kinetochore pairs from five cells were measured and the statistics analysis was performed using Excel software.

## Results

### Phosphorylation of Thr12 and Ser15 is dispensable for the kinetochore localization of Mps1

**Figure 1:**
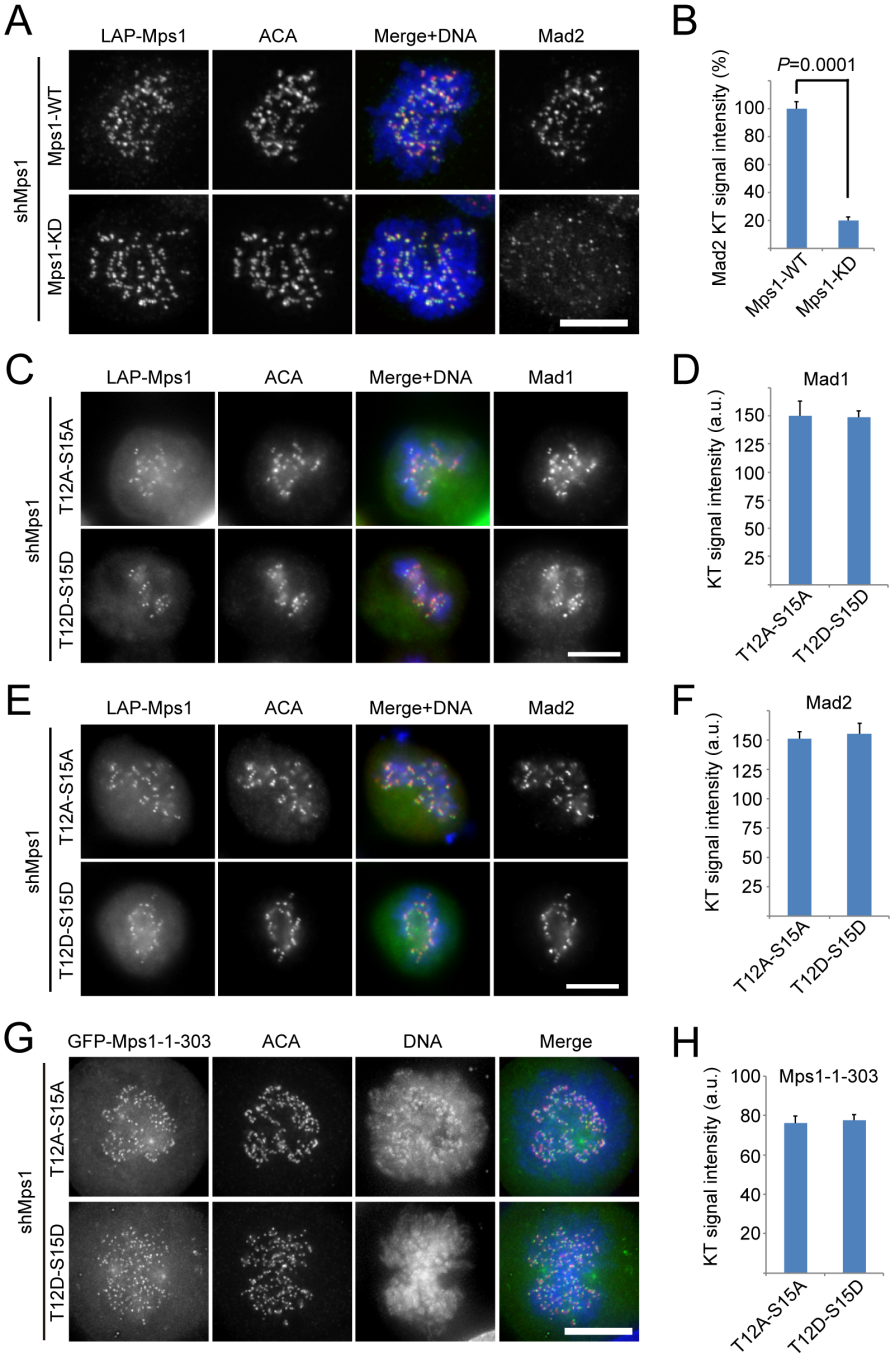
Autophosphorylation of Thr12 and Ser15 is dispensable for Mps1 kinetochore localization and SAC function. (A) Representative immunofluorescence images of prometaphase cells expressing different LAP-tagged Mps1 constructs. At 36 hours after co-transfection with the Mps1 shRNA and indicated plasmids, cells were fixed and co-stained for ACA (red), DNA (blue) and Mad2 (shown as gray scale images). (B) Bar graph showing quantification of the relative Mad2 kinetochore signal intensity in LAP-Mps1^WT^ or Mps1^KD^ expressing cells. Bars indicate mean ±SE from 5 cells measured (at least 20 kinetochores per cell). (C) and (E) Representative immunofluorescence images of prometaphase cells expressing different LAP-tagged Mps1 constructs. At 36 hours after co-transfection with the Mps1 shRNA and indicated plasmids, cells were fixed and co-stained for ACA (red), DNA (blue) and Mad1 (C, shown as gray scale images) or Mad2 (E, showing as gray scale images). Scale bar represents 10 µm. (D) and (F) Bar graph showing quantification of the Mad1 (D) or Mad2 (F) kinetochore signal in cells treated as in (C) or (E). Bars indicate mean ±SE from 5 cells measured (at least 20 kinetochores per cell). A.U. means arbitrary unit. (G) Representative immunofluorescence images of prometaphase cells expressing different GFP-tagged Mps1 truncations. At 36 hours after co-transfection with the Mps1 shRNA and indicated plasmids, cells were fixed and co-stained for ACA (red) and DNA (blue). Scale bar represents 10 µm. (H) Bar graph showing quantification of the kinetochore signal of indicated Mps1 truncation protein as in (G). Bars indicate mean ±SE from 5 cells measured (at least 20 kinetochores per cell).

During mitosis, Mps1 is hyperphosphorylated and has the maximum kinase activity [9]. A number of phosphorylation sites in Mps1 have been identified and many of them were shown to be autophosphorylation sites [26], [29]–[32], [40]. Xu *et al*. identified 9 sites as autophosphorylation sites, among which two autophosphorylation sites (Thr12, Ser15) within the kinetochore targeting region are required for the kinetochore recruitment of Mps1 [31]. As inhibition of Mps1 kinase activity significantly enhanced the kinetochore localization of Mps1 (Fig. S1), consistent with recent publications [12], [16], [28], it is unlikely that phosphorylation of Thr12 and Ser15 is required for the kinetochore localization of Mps1. It is also possible that the two phosphorylation sites are not genuine autophosphorylation sites. To confirm that Thr12 and Ser15 phosphorylation is not important for the localization of Mps1 and for the SAC, we examined the localization of two Mps1 mutants: nonphosphorylatable mutant Mps1^2A^ (the residues Thr12 and Ser15 were mutated to alanine) and phospho-mimetic mutant Mps1^2D^ (the residues Thr12 and Ser15 were mutated to aspartic acid), and their effect on the kinetochore localization of Mad1 and Mad2. In cells treated with Mps1 shRNA, Mad2 was not detected at kinetochore (Fig. S2). When knocking-down endogenous Mps1 and simultaneously expressing shRNA-resistent Mps1 wild type (WT), we found that Mad2 appeared again at kinetochore, whereas it remained nearly undetectable at kinetochore in Mps1 kinase-dead (KD) expressing cells (Fig. 1A and 1B). Similar findings were obtained when examining Mad1 (Fig. S3). Note that this is inconsistent with an early report showing that Mps1^KD^ can restore the kinetochore localization of Mad1 [41]. In the absence of endogenous Mps1, both Mps1^2A^ and Mps1^2D^ localized to kinetochore and restored the kinetochore localization of Mad1 and Mad2 (Fig. 1C, 1D, 1E and 1F). Since the fragment of amino acids 1–303 is sufficient for efficient kinetochore localization of Mps1, we further examined the localization of Mps1 truncations (amino acids 1–303) containing either the two nonphosphorylatable mutations (Mps1^1–303–2A^) or the two phospho-mimicking mutations (Mps1^1–303–2D^). As shown in Fig. 1G and 1H, both Mps1^1–303–2A^ and Mps1^1–303–2D^ localize to kinetochore, similar to Mps1^1–303^. Taken together, our findings suggest that phosphorylation of Thr12 and Ser15 is not required for the kinetochore localization of Mps1.

### Autophosphorylation releases Mps1 from kinetochore

**Figure 2:**
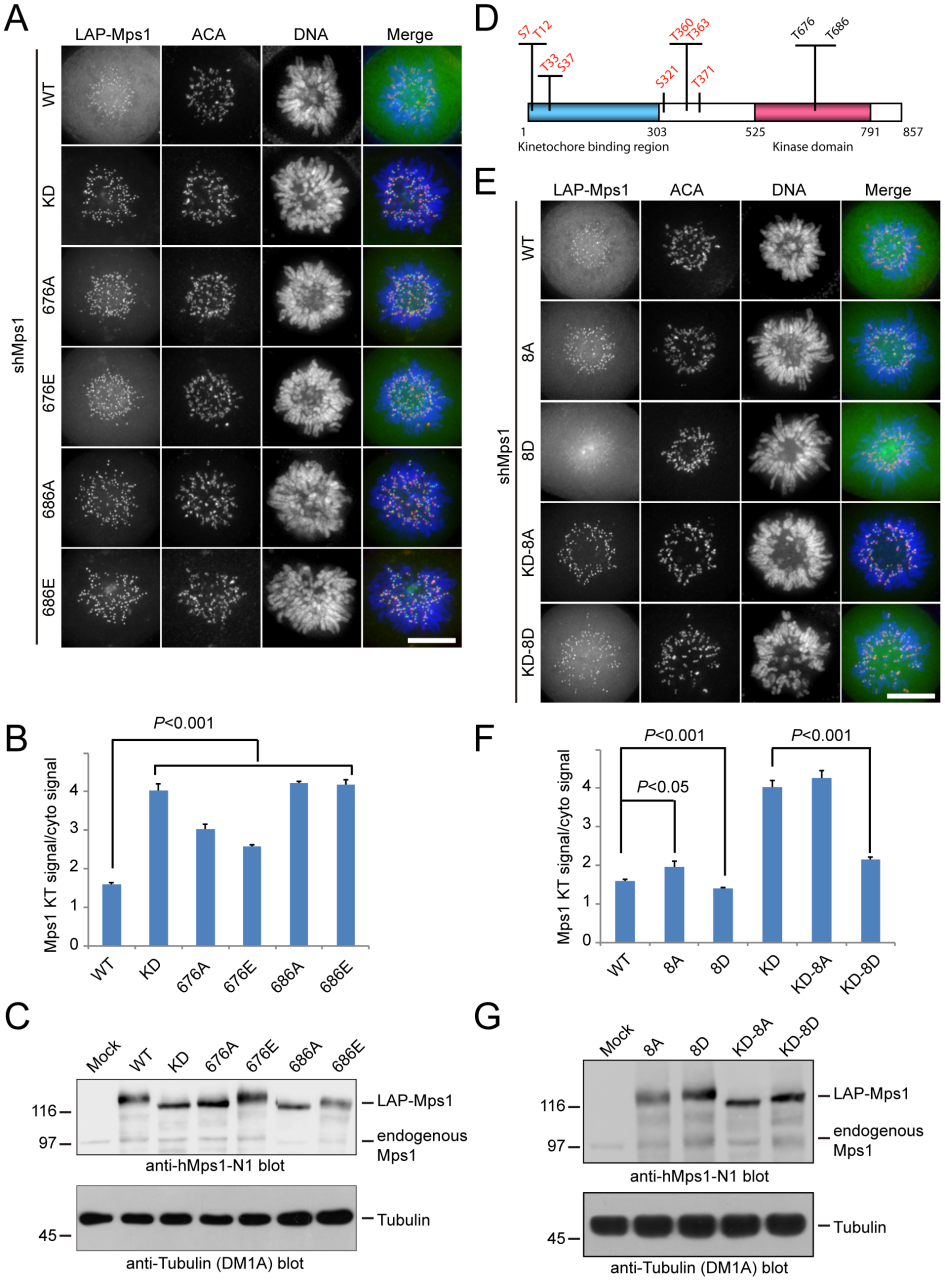
Autophosphorylation negatively regulates Mps1 kinetochore localization. (A) and (E) Representative immunofluorescence images of prometaphase cells expressing different LAP-tagged Mps1 constructs. At 36 hours after co-transfection with the Mps1 shRNA and indicated plasmids, cells were fixed and co-stained for ACA (red) and DNA (blue). Scale bar represents 10 µm. (B) and (F) Bar graph showing quantification of the ratios of kinetochore signal to cytoplasmic signal of different Mps1 constructs as indicated. Bars indicate mean ±SE from 3 independent experiments. In each experiment, 5 cells were measured (>60 kinetochores per cell). Statistics significance was determined by an unpaired Student's t test. (C) and (G) Western blot showing the comparable expression of different Mps1 constructs as indicated. 24 hours after transfection into 293T cells, cell lysates were prepared. After separation by SDS-PAGE, samples were probed with the indicated antibodies. (D) Schematic showing Mps1 autophosphorylation sites. 8 autophosphorylation sites outside of kinase domain are shown in red. 2 autophosphorylation sites within activation loop are shown in black.

Consistent with the earlier publications [12], [16], we observed much stronger kinetochore localization for Mps1^KD^ than Mps1^WT^ (Fig. 2A). Therefore, Mps1 kinase activity may negatively regulate the kinetochore localization of Mps1. In general, the residues within the activation loop of a kinase and their posttranslational modification (mainly phosphorylation) are critical for the fully activation of a protein kinase [42]. Previous studies showed that Mps1^T676A^ has less active kinase activity than Mps1^WT^ [29]–[30]. In addition, phospho-mimetic Mps1^T676E^ is also less-active than Mps1^WT^. Both phospho-deficient and phospho-mimetic mutants of Thr686, another key phosphorylation site within the kinase P+1 loop, are completely inactive [29]–[30]. To further test our hypothesis that the kinetochore localization of Mps1 may be negatively regulated by its kinase activity, we examined the localization of these mutants: Mps1^T676A^, Mps1^T676E^, Mps1^T686A^ and Mps1^T686E^. As shown in Fig. 2A, both Mps1^T676A^ and Mps1^T676E^ displayed strong kinetochore staining with moderate cytoplasmic signals. Similarly, both Mps1^T686A^ and Mps1^T686E^ displayed strong kinetochore staining but had faint staining in the cytoplasm, indistinguishable from Mps1^KD^ (Fig. 2A and 2B). As all these Mps1 mutants were expressed at a level comparable to Mps1^WT^ (Fig. 2C), we concluded that the different kinetochore staining observed was not due to variable protein expression levels. Our observation that the completely inactive mutants Mps1^T686A^ and Mps1^T686E^ showed stronger kinetochore signals than the less-active mutants Mps1^T676A^ and Mps1^T676E^ suggests that the kinetochore localization of Mps1 is negatively regulated by its own kinase activity.

It is possible that Mps1 regulates its own kinetochore localization by phosphorylating an as yet unknown kinetochore substrate that is responsible for recruiting Mps1. Alternatively, Mps1 inhibits its localization by autophosphorylation. If the latter case is true, one would expect to see stronger kinetochore staining for phospho-deficient autophosphorylation mutant compared to the phospho-mimetic autophosphorylation mutant. It has been shown that many phosphorylation sites of Mps1 are autophosphorylated [26], [30]–[31]. To test if autophosphorylation plays a critical role in regulating the kinetochore localization of Mps1, we created LAP-tagged Mps1 mutants that were either phospho-deficient (Mps1^8A^) or phospho-mimetic (Mps1^8D^) for the 8 autophosphorylation sites (Ser7, Thr12, Thr33, Ser37, Ser321, Thr360, Thr363 and Thr371) that conform to our proposed Mps1 consensus motif E/D/N/Q-X-pS/pT-X (Fig. 2D) [26]. As shown (Fig. 2E), we observed elevated kinetochore signal intensity of Mps1^8A^ and decreased kinetochore signal intensity of Mps1^8D^. Interestingly, the kinetochore staining of Mps1^8A^ was still weaker than that of Mps1^KD^. These observations suggest that autophosphorylation is required for the release of Mps1 from kinetochore.

Further, we made Mps1^8A^ and Mps1^8D^ become kinase-dead by mutagenesis and examined their localization. If autophosphorylation is required for the release of Mps1 from kinetochore, one would expect to see a weaker kinetochore staining for the autophosphorylation mimetic Mps1^KD–8D^ than for the autophosphorylation deficient Mps1^KD–8A^ mutant. Consistently, Mps1^KD–8D^ staining at kinetochores became much weaker and displayed strong straining in the cytoplasm (Fig. 2E). In contrast, Mps1^KD–8A^ and Mps1^KD^ localized mainly to kinetochores. Quantification of the ratio of kinetochore/cytosol signal intensity for each mutant also confirmed our observations (Fig. 2F). Similar results were obtained when examining the phospho-mutants lacking the kinase domain (Fig. S4). Note that all the mutants examined were expressed at a comparable level (Fig. 2G). Thus, we conclude that autophosphorylation of Mps1 facilitates the kinetochore release of Mps1.

### Autophosphorylation of Mps1 at its N-terminal region does not affect its kinase activity

Generally, autophosphorylation in the activation loop of Mps1 is required for full kinase activity [30], [32]–[33]. We, therefore, asked whether autophosphorylation taking place at the region outside of the kinase domain can influence Mps1 kinase activity. To test this, we carried out an *in vitro* kinase assay using recombinant Borealin as substrate with approximate the same amount of different immunoprecipitated Mps1 proteins (Fig. S5A) [15]. As shown (Fig. S5B), Mps1^WT^, not Mps1^KD^, could phosphorylate Borealin. Similar to Mps1^WT^, both autophosphorylation site mutants Mps1^8A^ and Mps1^8D^ could efficiently phosphorylate Borealin (Fig. S5B), suggesting that autophosphorylation of the N-terminal region (at least at the sites described here) does not affect Mps1 kinase activity. Consistent with the previous report that CDK1 potentiate Mps1 activity [43],the positive control phospho-mimetic mutant Mps1^5D^ (5 CDK1 phosphorylation sites S281, S436, T453, T468, S821) used in these assays displayed enhanced kinase activity.

Interestingly, we observed two separated phosphorylation species in the Mps1 immuno-precipitates and the kinase assays (Fig. S5B). Two possibilities may account for this observation: 1) both bands represent Mps1, but they are phosphorylated to different degrees; and 2) only one band is Mps1 and the other is a contamination from either kinase immunoprecipitates or substrate preparation. For clarification, we performed an *in vitro* kinase assay with recombinant GST-Mps1, but without Borealin. GST-Mps1 displayed two clearly separated bands (Fig. S5C). The upper band was not affected by Plk1 kinase inhibitor TAL, but disappeared completely in the presence of Reversine, suggesting that the upper autoradiography band represents a highly phosphorylated pool of Mps1. Moreover, Mps1^8A^, which lacks 8 autophosphorylation sites, does not shift to the same extent as Mps1^WT^, further supporting the idea that the upper band corresponds to Mps1 (Fig. S5B). As only very weak bands at the position of upper autoradiography band were detected by Western blotting (Fig. S5B), we proposed that upper autoradiography band only accounts for a small proportion of the total Mps1, or that the modifications preclude visualization of this band by Western blotting.

### Dynamic phosphorylation of Mps1 is required for proper mitotic progression

**Figure 3:**
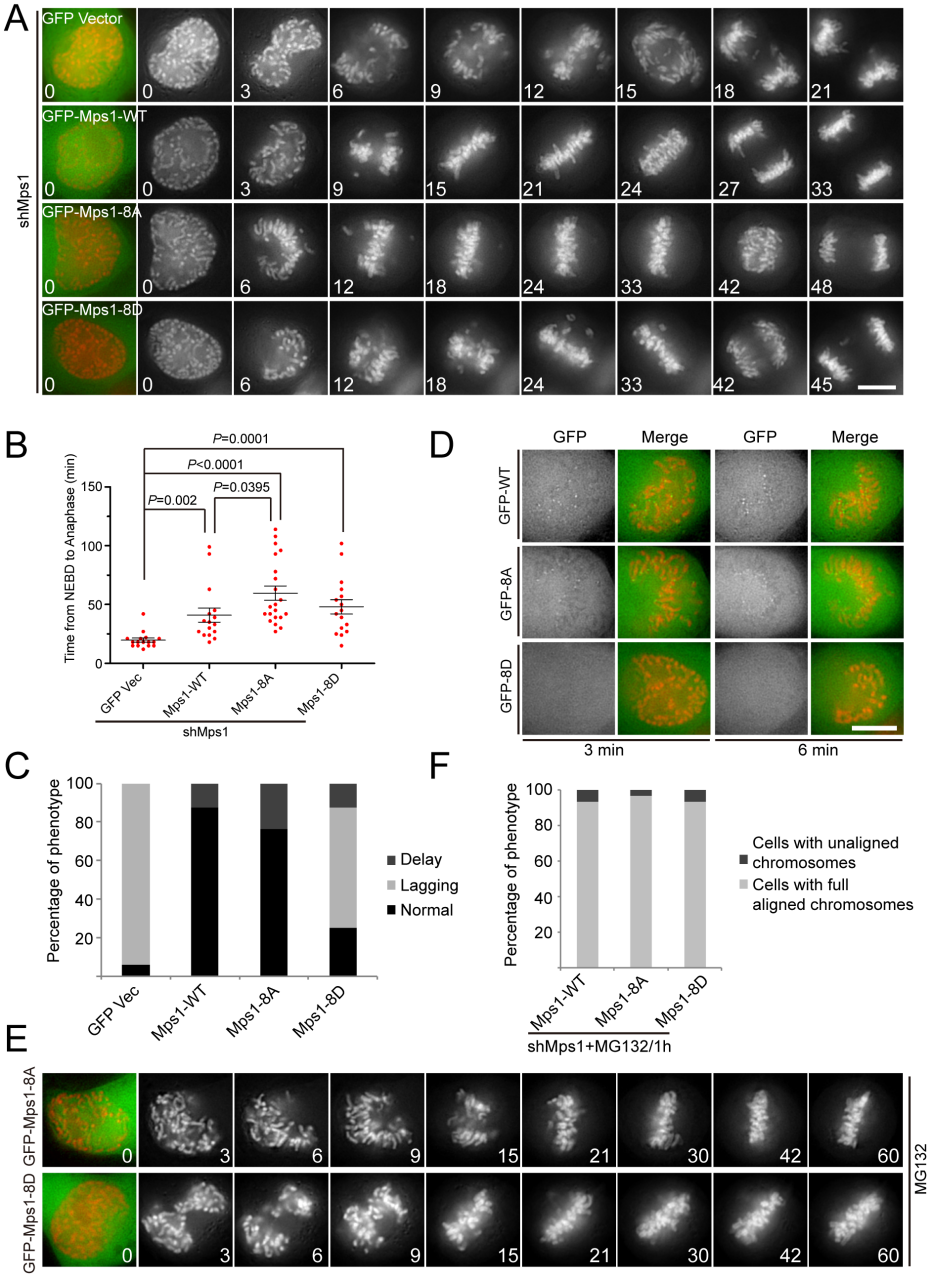
Characterizing the mitotic phenotype of phospho-defective and phospho-mimetic mutants of autophosphorylation sites. (A) and (D) Representative stills illustrating mitotic progression in H2B-mCherry expressing cells depleted of Mps1 and rescued with different LAP-Mps1 constructs. Images were acquired at the indicated time points after the start of nuclear envelope breakdown (NEB). Scale bar represents10 µm. (B) Scatter plots indicating the time elapsed from NEB to anaphase onset with the mean in cells treated as in A. Bars indicate mean ±SE from analyses of at least 16 cells. Statistics significance was determined by an unpaired Student's t test. (C) Bar graph showing quantification of the mitotic progression phenotype of cells expressing indicated LAP-Mps1 constructs. “Delay” means mitotic delay (last more than 90 minutes to enter anaphase). “Lagging” means the anaphase cells with lagging chromosome or unaligned chromosome. (E) Representative stills illustrating mitotic progression in H2B-mCherry expressing cells depleted of Mps1 and rescued with different LAP-Mps1 constructs. Before imaging, the cells were treated with MG132. Images were acquired at the indicated time points after the start of nuclear envelope breakdown (NEB). (F) Bar graph showing the percentage of indicated phenotype of cells treated as in (E). 30 cells were counted in each group.

Next, we employed live cell imaging to examine if autophosphorylation of Mps1 contributes to regulating mitotic progression. The absence of endogenous Mps1 allowed cells to prematurely enter anaphase and caused a defect in chromosome congression (Fig. 3A). Ectopic expression of Mps1^WT^ in cells lacking endogenous Mps1 restored normal mitotic progression and chromosome congression. Interestingly, ectopic expression of Mps1^8A^ in cells lacking endogenous Mps1 also restored normal mitotic progression and chromosome congression, but delayed anaphase entry (Fig. 3B). In Mps1^8D^ expressing cells, we observed delayed anaphase entry, chromosome congression errors, and anaphase lagging chromosomes (Fig. 3A, 3B and 3C), suggesting that the SAC in these cells have been compromised. Consistently, although the kinetochore localization of Mps1^WT^ and Mps1^8A^ were clearly visible in the live cell imaging condition, Mps1^8D^ was difficult to see at kinetochores (Fig. 3D). Therefore, the compromised SAC in Mps1^8D^ expressing cells may be due to decreased kinetochore localization of Mps1^8D^. In addition, the enhanced kinetochore localization of Mps1^8A^ may contribute to the delayed anaphase entry. Next, we examined carefully to see if Mps1^8A^ and Mps1^8D^ also affect metaphase chromosome congression, employing the drug MG132. In the presence of MG132, both Mps1^8A^ and Mps1^8D^ expressing cells could be arrested at metaphase but did not show defects in metaphase chromosome congression (Fig. 3E and 3F). Despite normal chromosome congression, the high frequency of anaphase lagging chromosome observed in Mps1^8D^ expressing cells suggests that the correction of merotelic attachment is defective in these cells. Taken together, our data suggest that dynamic autophosphorylation of Mps1 is required for proper mitotic progression timing but not for metaphase chromosome alignment.

### Persistent autophosphorylation of Mps1 causes a decreased kinetochore localization of BubR1 and Mad2

**Figure 4:**
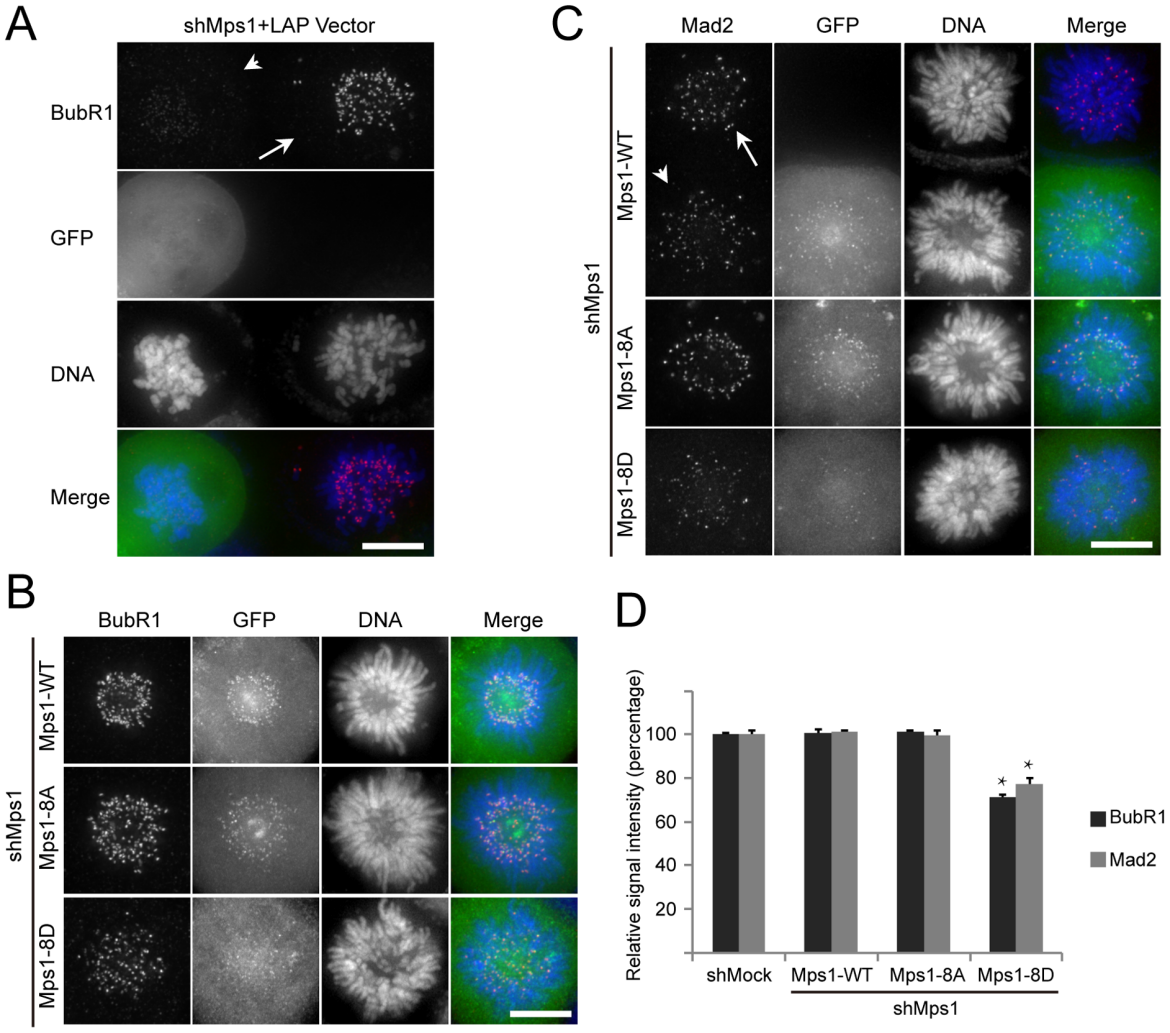
Phospho-mimetic Mps1^8D^ impairs the kinetochore recruitment of BubR1 and Mad2. (A) Representative immunofluorescence images of prometaphase cells co-transfected with Mps1 shRNA and GFP vector at a 3∶1 ratio. Cells were fixed and co-stained for BubR1 (red) and DNA (blue). Scale bar represents 10 µm. (B) and (C) Representative immunofluorescence images of prometaphase cells expressing different LAP-tagged Mps1 constructs. At 36 hours after co-transfection with the Mps1 shRNA and indicated plasmids, cells were fixed and co-stained for BubR1 (red) (B) or Mad2 (C), and DNA (blue). Scale bar represents 10 µm. (D) Bar graph showing quantification of kinetochore signal of BubR1 and Mad2 in cells expressing different Mps1 constructs as indicated. Bars indicate mean ±SE from 3 independent experiments. In each experiment, 5 cells were measured (>60 kinetochores per cell). * *P*<0.001 versus shMock or Mps1^WT^ and Mps1^8A^ rescue groups.

To understand how Mps1^8D^ compromises SAC function, we examined the kinetochore localization of two SAC effector proteins: BubR1 and Mad2. In agreement with previous publications [13] [44], BubR1 kinetochore signals decreased significantly in Mps1 shRNA transfected cells (Fig. 4A, arrowhead), as compared to the control cells (Fig. 4A, arrow). When examining Mps1^8A^ and Mps1^WT^ expressing cells, respectively, we found that the kinetochore intensity of BubR1 in these two cells was comparable (Fig. 4B). However, the kinetochore intensity of BubR1 decreased significantly in Mps1^8D^ expressing cells (See Fig. 4B and 4C for statistical analysis). In cells lacking Mps1, expression of Mps1^WT^ restored the kinetochore localization of Mad2. As shown (Fig. 4C), in an Mps1^WT^-expressing cell (arrowhead) and its adjacent untransfected cell (arrow), the Mad2 kinetochore intensity was indistinguishable. Similarly, Mad2 intensity in Mps1^8A^-expressing cells was at a level approximately equal to the Mad2 intensity in Mps1^WT^ expressing cells. In contrast, the Mad2 signal intensity decreased significantly in Mps1^8D^-expressing cells (Fig. 4C, 4D). Thus, our data suggest that Mps1^8D^ compromises the SAC by affecting the kinetochore recruitment of BubR1 and Mad2.

## Discussion

Autophosphorylation at the activation loop is required for Mps1 activation [32]–[33]. Our previous study demonstrated that 8 phosphorylation sites outside of the activation loop are autophosphorylation sites [26]. Here, we demonstrate that these autophosphorylation sites that are not present in the activation loop are involved in regulating the kinetochore localization of Mps1. Autophosphorylation of these sites is required for the release of Mps1 from kinetochore.

We provide several pieces of evidence to support our claim. First, phospho-mimicking mutations of the 8 autophosphorylation sites (Mps1^8D^) cannot localize to kinetochore as efficiently as the nonphosphorylatable Mps1 mutant Mps1^8A^ and Mps1^WT^ (Fig. 2E). Since multi-site phosphorylation is now recognized as an important mechanism for attenuation of protein-protein and protein-ligand interactions [45], it is possible that autophosphorylation may attenuate the interaction between Mps1 and Hec1 or between Mps1 and its kinetochore adaptor proteins, thus causing a kinetochore localization defect. Consistently, Jelluma et al. reported that Mps1 is rapidly exchanged at unaligned kinetochores by a mechanism involved Mps1-dependent phosphorylation [28]. Second, Mps1^KD^ displayed much stronger kinetochore staining than Mps1^WT^ and even the less active Mps1 mutants (Mps1^T676A^, Mps1^T676E^) exhibited stronger kinetochore staining than Mps1^WT^ (Fig. 2A). We reason that Mps1^KD^ and the less active Mps1 mutants may not undergo autophosphorylation or at least less efficiently, which then enhances their kinetochore localization.

Intriguingly, the kinetochore localization of Mps1^8A^ is significantly weaker than Mps1^KD^, but clearly stronger than Mps1^WT^ or Mps1^8D^. Mps1^WT^, Mps1^8A^ and Mps1^8D^, except Mps1^KD^, have comparable kinase activity (Fig. S5B). This means that Mps1^WT^, Mps1^8A^ and Mps1^8D^ are capable of undergoing autophosphorylation. We speculate that more autophosphorylation sites may exist, thus allowing Mps1^8A^ to be partially autophosphorylated. This partial phosphorylation may then promote the release of Mps1^8A^ from the kinetochore. Therefore, Mps1^8A^ displays weaker kinetochore localization than Mps1^KD^, but stronger than Mps1^WT^ or Mps1^8D^.

Mps1^8A^ causes shortly delayed anaphase entry. This may be due to the enhanced and less dynamic kinetochore localization, which is supported by the observation that tethering Mps1 to kinetochore by expressing Mis12-Mps1 fusion protein similarly prolongs metaphase [28]. In contrast, Mps1^8D^ causes premature anaphase entry with misaligned chromosomes and/or anaphase lagging chromosomes. We reason that the phenotypes observed in Mps1^8D^ expressing cells could due to 1) the compromised SAC and 2) defective correction of merotelic attachment. The compromised SAC is well supported by the fact that two key SAC effectors, BubR1 and Mad2, display reduced kinetochore localization in Mps1^8D^ expressing cells (Fig. 4D). It has been proposed that instead of an all-or-none response, SAC signal strength varies in different conditions [46]–[48]. These studies also revealed that SAC strength correlates with the Mad2 signal intensity at kinetochore. Therefore, Mps1^8D^ expressing cells may not lose the SAC completely, but have compromised SAC. We emphasize that the SAC is likely to be affected slightly in Mps1^8D^ expressing cells, since the Mad2/BubR1 kinetochore signal decreased slightly. Thus, in Mps1^8D^ expressing cells, the SAC is able to prevent anaphase entry for a comparable time span with Mps1^WT^ expressing cells.

As Mps1^8A^ and Mps1^8D^ expressing cells display proper chromosome bi-orientation in the presence of MG132 treatment (Fig. 3E, 3F), the 8 autophosphorylation sites studied here may not be involved in metaphase chromosome congression. Although chromosome congression is normal in the presence of MG132, the high frequency of lagging chromosome observed in Mps1^8D^ expressing cells indicates the presence of uncorrected merotelic attachments. Recent evidence suggests that Mps1 plays a key role in the correction of merotelic attachment [49]. Consistent with this notion, decreased kinetochore localization of Mps1^8D^ may induce merotelic attachments. The other possibility is the expression of Mps1^8D^ causes an additional hypomorphic effect on K-fiber integrity.

A recent report suggested that Mps1 kinetochore targeting requires its kinase activity and autophosphorylation at Thr12 and Ser15 [31]. However, our data show that both phospho-deficient and phospho-mimetic Thr12 and Ser15 double mutants (Mps1^2A^ and Mps1^2D^, respectively) localized to kinetochore normally and that both mutants do not affect the kinetochore localization of Mad1 and Mad2 (Fig. 1C and 1E). It is likely that Mps1 has more (auto)phosphorylation sites besides the 8 autophosphorylation sites we studied here. For example, Chk2 can phosphorylate Mps1 at Thr288 and this is required for the kinetochore localization of Mps1 [50]–[51]. Thus, a new theme for systematic identification and characterization of all phosphorylation sites in Mps1 is emerging.

In summary, we demonstrate that autophosphorylation is required for the release of Mps1 from kinetochore and is involved in regulating the kinetochore localization of SAC components BubR1 and Mad2. Autophosphorylation-deficient Mps1 mutant induces a shortly delayed mitotic progression and autophosphorylation-mimicking mutant perturbs faithful chromosome segregation. Together, these data indicate that dynamic autophosphorylation of Mps1 ensures accurate chromosome segregation and faithful mitotic progression.

## Supporting Information

Figure S1 **Mps1 kinetochore localization elevated greatly when its kinase activity was inhibited.** (A) Representative immunofluorescence images of prometaphase cells stably expressing LAP-tagged Mps1. At 2 hours after treatment with the indicated drugs, cells were fixed and co-stained for Mps1 (green), ACA (red), DNA (blue). Scale bar represents 10 µm. (B) Bar graph showing quantification of the kinetochore signal of cells treated as indicated. Bars indicate mean ±SE from 3 independent experiments. In each experiment, 5 cells were measured (>60 kinetochores per cell). a. u. means arbitrary unit.
(TIF)

Figure S2 **Mps1 kinase is required for the kinetochore recruitment of Mad2.** Representative immunofluorescence images of prometaphase cells transfected with Mps1 shRNA and Mock shRNA. At 36 hours after transfection, cells were fixed and co-stained for Mad2 (green), ACA (red), DNA (blue). Scale bar represents 10 µm.
(TIF)

Figure S3 **Mps1 kinase activity is stringently required for the kinetochore recruitment of Mad1.** Representative immunofluorescence images of prometaphase cells transfected with Mps1 shRNA and different LAP-Mps1 constructs as indicated. At 36 hours after transfection, cells were fixed and co-stained for Mps1 (green), ACA (red), DNA (blue) and Mad1 (shown as gray scale images). Scale bar represents 10 µm.
(TIF)

Figure S4 **Autophosphorylation negatively regulate the kinetochore localization of Mps1 fragment lacking kinase domain.** (A) Representative immunofluorescence images of prometaphase cells transfected with Mps1 shRNA and different GFP-Mps1 constructs as indicated. At 36 hours after transfection, cells were fixed and co-stained for Mps1 (green), ACA (red) and DNA (blue). Scale bar represents 10 µm. (B) Bar graph showing quantification of the kinetochore signal of different Mps1 truncations as indicated. Bars indicate mean ±SE from 3 independent experiments. In each experiment, 5 cells were measured (>60 kinetochores per cell). a. u. means arbitrary unit.
(TIF)

Figure S5 **Mps1 autophosphorylation doesn't affect its kinase activity** ***in vitro*** **.** (A) Immunoblot (anti-hMps1) showing the correct expression of wild type Mps1 and different Mps1 mutants and equal amount of input lysate for each immunoprecipitation reaction. (B) *In vitro* kinase assay of recombinant MBP-tagged Borealin by LAP-tagged wild type and different Mps1 mutants. LAP-Mps1 transfected HeLa S3 cell were harvested and cell lysates were incubated with anti-hMps1-N1 mAb coupled protein G beads. Then the beads were incubated with MBP-Borealin in the presence of γ-^32^P-ATP. The left panel shows Coomassie Blue staining of the gel (asterisk indicates an unspecific band from MBP-Borealin purification), the right panel shows the autoradiography result. The lower panel shows the anti-hMps1 blot of the kinase inputs used in different reactions. (C) *In vitro* kinase assay of recombinant GST-Mps1-WT in kinase buffer with DMSO, buffer with TAL and buffer with Reversine. The upper panel shows the autoradiography result; the lower panel shows the anti-hMps1 Western blot demonstrating equal loading.
(TIF)
