# Supplementary figures and images for: Dynamic Autophosphorylation of Mps1 Kinase Is Required for Faithful Mitotic Progression
Source: PLoS One. 2014 Sep 29;9(9):e104723. doi: 10.1371/journal.pone.0104723 (PMC4179234; doi:10.1371/journal.pone.0104723)

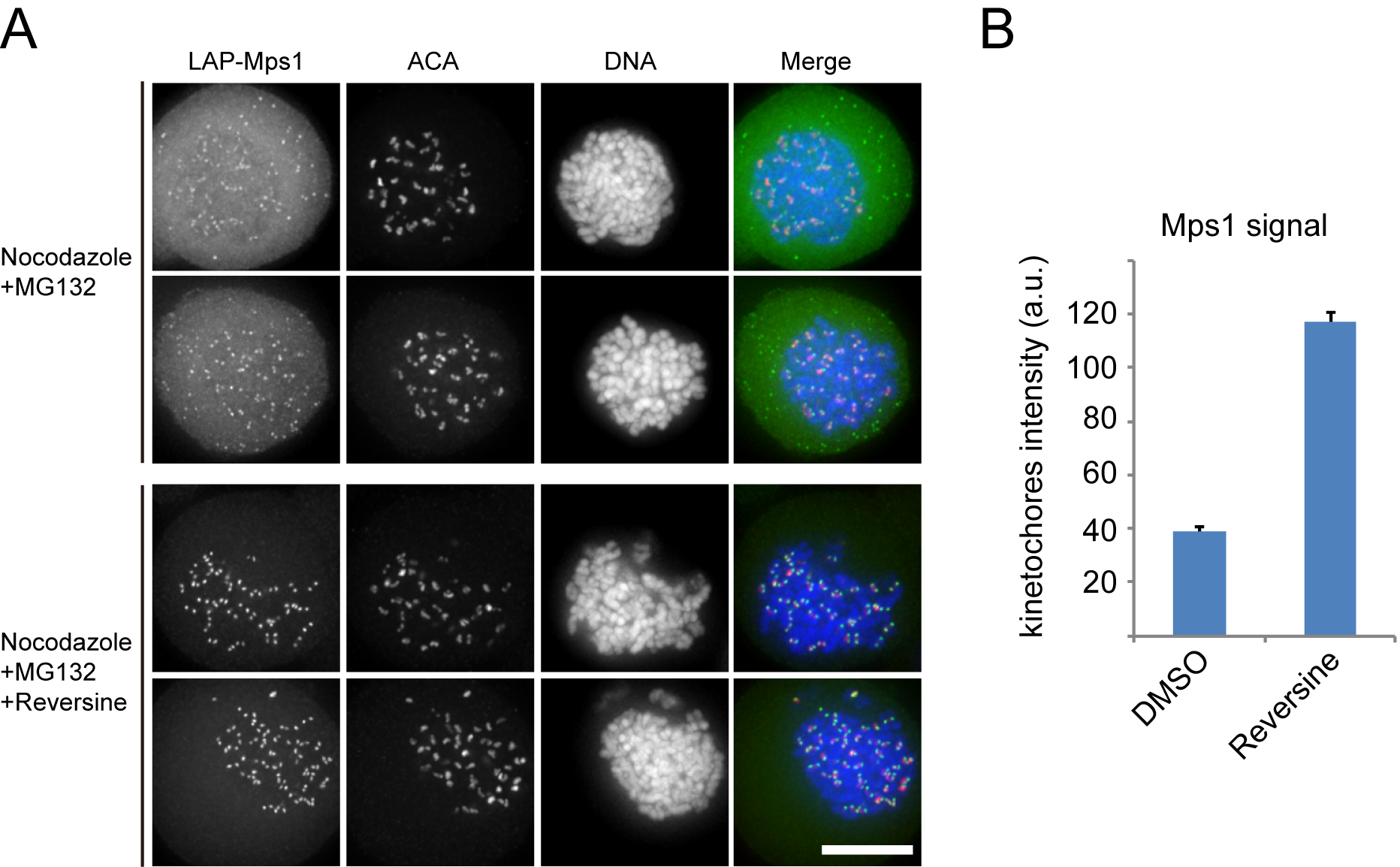

Supplement: Figure S1 — Mps1 kinetochore localization elevated greatly when its kinase activity was inhibited. (A) Representative immunofluorescence images of prometaphase cells stably expressing LAP-tagged Mps1. At 2 hours after treatment with the indicated drugs, cells were fixed and co-stained for Mps1 (green), ACA (red), DNA (blue). Scale bar represents 10 µm. (B) Bar graph showing quantification of the kinetochore signal of cells treated as indicated. Bars indicate mean ±SE from 3 independent experiments. In each experiment, 5 cells were measured (>60 kinetochores per cell). a. u. means arbitrary unit. (TIF) [file pone.0104723.s001.tif]

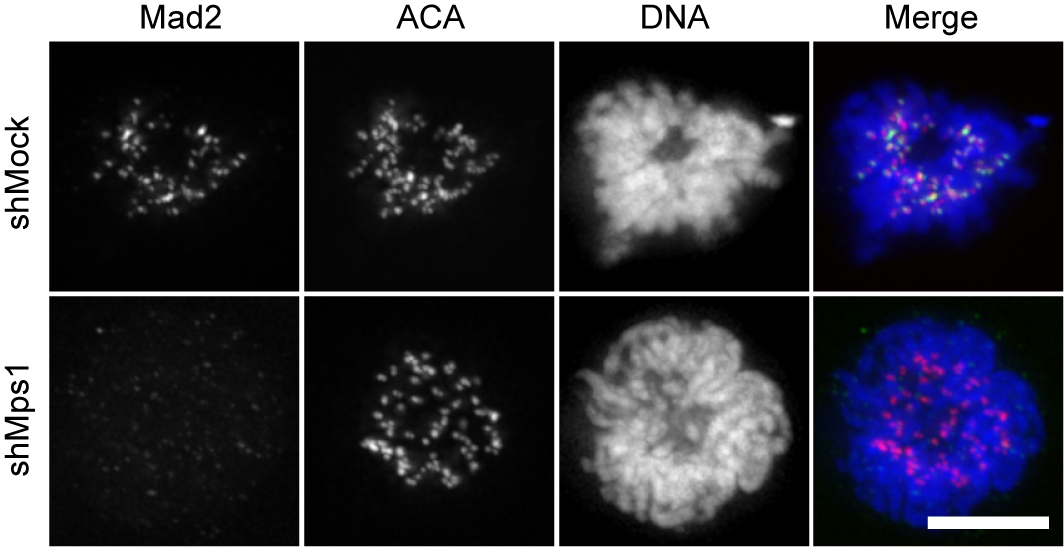

Supplement: Figure S2 — Mps1 kinase is required for the kinetochore recruitment of Mad2. Representative immunofluorescence images of prometaphase cells transfected with Mps1 shRNA and Mock shRNA. At 36 hours after transfection, cells were fixed and co-stained for Mad2 (green), ACA (red), DNA (blue). Scale bar represents 10 µm. (TIF) [file pone.0104723.s002.tif]

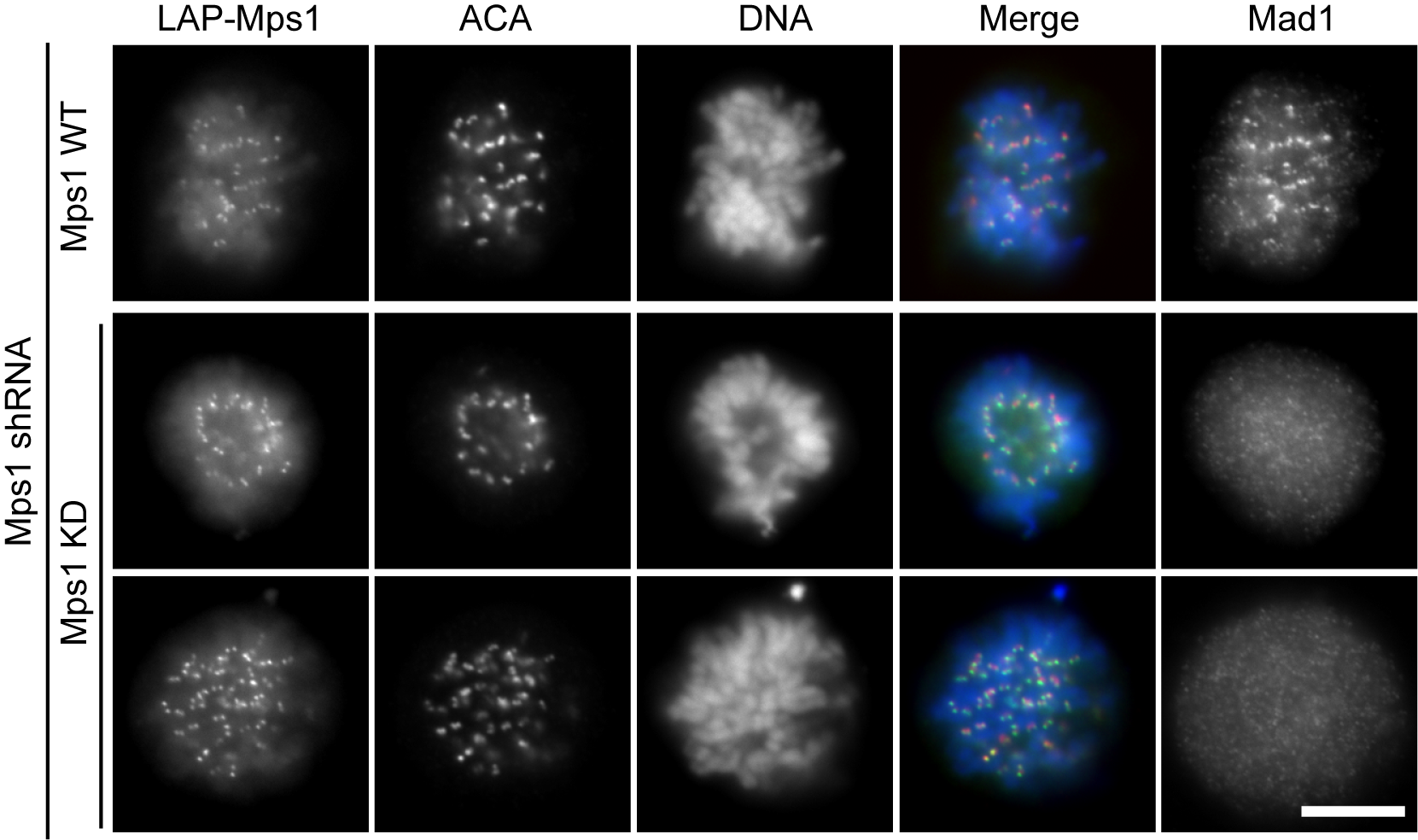

Supplement: Figure S3 — Mps1 kinase activity is stringently required for the kinetochore recruitment of Mad1. Representative immunofluorescence images of prometaphase cells transfected with Mps1 shRNA and different LAP-Mps1 constructs as indicated. At 36 hours after transfection, cells were fixed and co-stained for Mps1 (green), ACA (red), DNA (blue) and Mad1 (shown as gray scale images). Scale bar represents 10 µm. (TIF) [file pone.0104723.s003.tif]

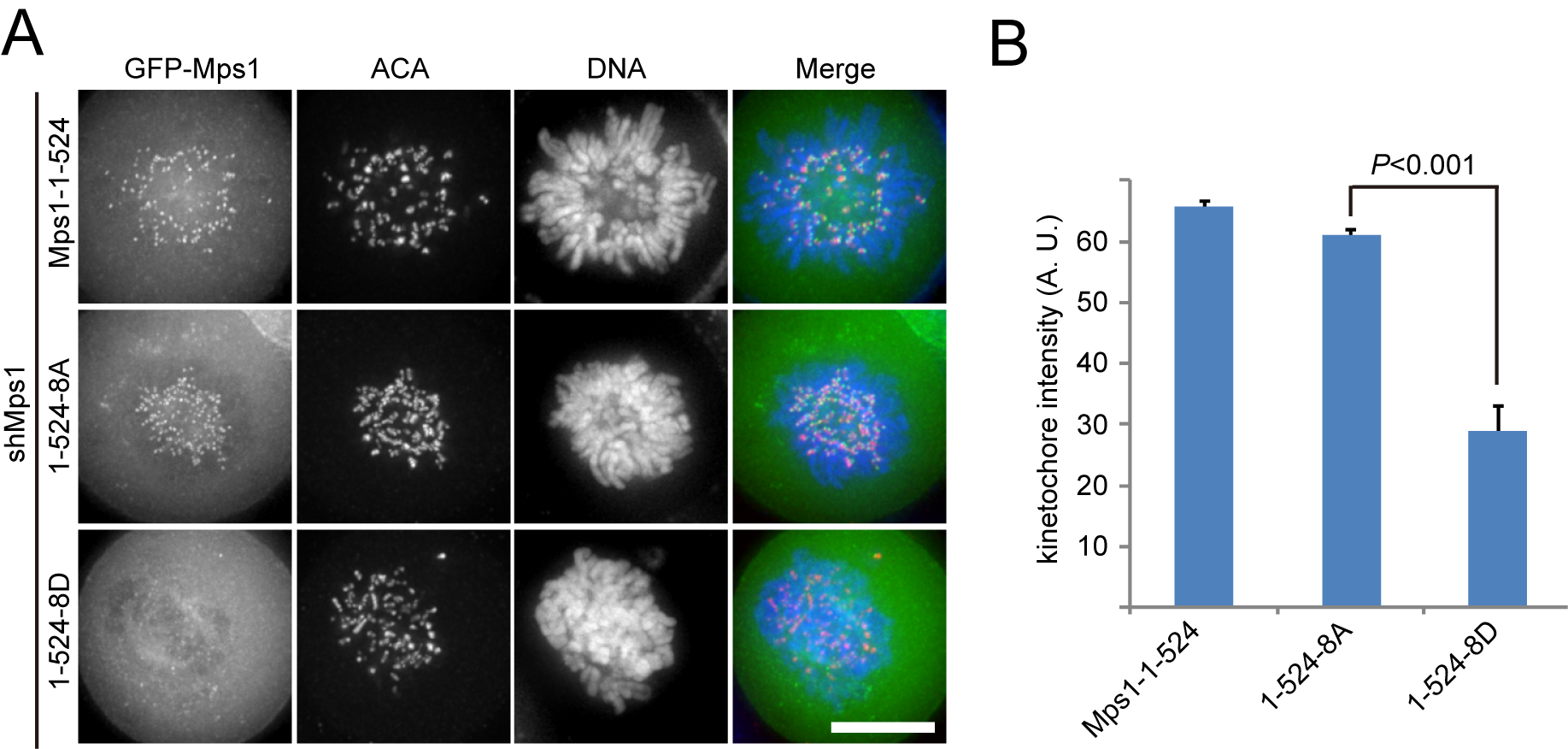

Supplement: Figure S4 — Autophosphorylation negatively regulate the kinetochore localization of Mps1 fragment lacking kinase domain. (A) Representative immunofluorescence images of prometaphase cells transfected with Mps1 shRNA and different GFP-Mps1 constructs as indicated. At 36 hours after transfection, cells were fixed and co-stained for Mps1 (green), ACA (red) and DNA (blue). Scale bar represents 10 µm. (B) Bar graph showing quantification of the kinetochore signal of different Mps1 truncations as indicated. Bars indicate mean ±SE from 3 independent experiments. In each experiment, 5 cells were measured (>60 kinetochores per cell). a. u. means arbitrary unit. (TIF) [file pone.0104723.s004.tif]

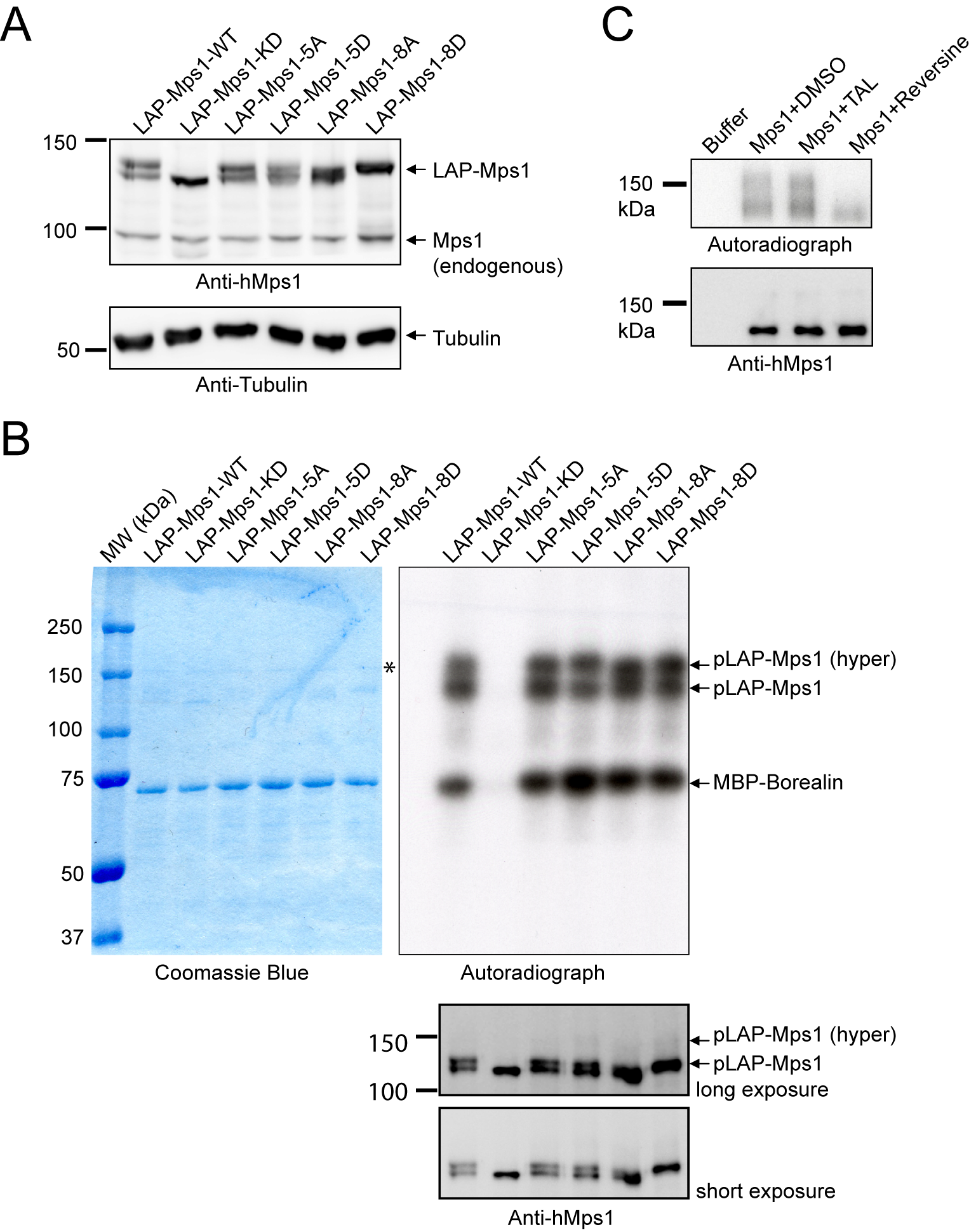

Supplement: Figure S5 — Mps1 autophosphorylation doesn't affect its kinase activity in vitro . (A) Immunoblot (anti-hMps1) showing the correct expression of wild type Mps1 and different Mps1 mutants and equal amount of input lysate for each immunoprecipitation reaction. (B) In vitro kinase assay of recombinant MBP-tagged Borealin by LAP-tagged wild type and different Mps1 mutants. LAP-Mps1 transfected HeLa S3 cell were harvested and cell lysates were incubated with anti-hMps1-N1 mAb coupled protein G beads. Then the beads were incubated with MBP-Borealin in the presence of γ-32P-ATP. The left panel shows Coomassie Blue staining of the gel (asterisk indicates an unspecific band from MBP-Borealin purification), the right panel shows the autoradiography result. The lower panel shows the anti-hMps1 blot of the kinase inputs used in different reactions. (C) In vitro kinase assay of recombinant GST-Mps1-WT in kinase buffer with DMSO, buffer with TAL and buffer with Reversine. The upper panel shows the autoradiography result; the lower panel shows the anti-hMps1 Western blot demonstrating equal loading. (TIF) [file pone.0104723.s005.tif]
